# Stereopsis Assessment in a Pediatric Population and Its Association With Refractive Error: A Cross-Sectional Study

**DOI:** 10.7759/cureus.108877

**Published:** 2026-05-15

**Authors:** Kanishk Singh, Rajesh Pattebahadur, Sagarika Snehi

**Affiliations:** 1 Ophthalmology, All India Institute of Medical Sciences, Nagpur, Nagpur, IND

**Keywords:** anisometropia, binocular vision, isometropia, refractive error, stereopsis

## Abstract

Introduction: Stereopsis is the perception of depth arising from binocular vision and is essential for fine motor skills and overall visual function in children. It has been observed that refractive errors diminish binocular function by causing visual blur and impairing fusion, which results in poor stereopsis. Poor stereoacuity can lead to inferior quality of vision and substandard work performance, especially where good eye-hand coordination and visual motor skills are required.

Purpose: The purpose of this study is to assess the level of stereoacuity among children with refractive errors and to determine the prevalence and pattern of subnormal stereopsis in the pediatric population.

Material and methods: This cross-sectional study was conducted at a tertiary care center in Central India from October 2023 to September 2024 after Institutional Ethics Committee approval. A total of 120 children aged 5-17 years with previously diagnosed refractive errors and a history of spectacle use for at least one month were included. Children with strabismus, amblyopia, ocular pathology, or poor cooperation were excluded. All participants underwent detailed ophthalmic evaluation, including visual acuity assessment, slit-lamp examination, fundus evaluation, and refraction (dry and cycloplegic), followed by subjective correction. Stereoacuity was assessed using the Randot stereotest and categorized as normal (10-60 arc seconds), subnormal (61-120 arc seconds), moderate (121-240 arc seconds), and poor (≥241 arc seconds). Anisometropia was defined as an interocular difference of ≥ 1 D spherical or ≥ 1.5 D cylindrical power. Statistical analysis was performed using Statistical Package for the Social Sciences (SPSS, version 17.0; SPSS Inc., Chicago, IL), and associations were tested using the chi-square test, with p < 0.05 considered significant.

Results: The study included 120 children with a mean age of 11 ± 2.64 years, comprising 65 (54.17%) males and 55 (45.83%) females. The mean stereoacuity was 54.01 ± 32.19 arc seconds. Anisometropia was present in 19 (15.83%) patients. Normal stereopsis was observed in 96.82% of myopic patients, 93.48% of astigmatic patients, and 72.73% of hyperopic patients. Moderate-to-poor stereopsis was predominantly seen in hyperopic individuals (27.27%), whereas only 3.18% of myopes demonstrated subnormal stereopsis, with none showing moderate or severe impairment. Hypermetropic children had significantly poorer stereopsis compared to myopes. Anisometropia was associated with reduced stereopsis. Isometropic individuals demonstrated better stereoacuity (48 ± 28.18 arc seconds) compared to anisometropic individuals (60 ± 36.89 arc seconds) (t = 2.31, p = 0.02), indicating a significant impact of on binocular function.

Conclusion: Hypermetropia is associated with poorer stereoacuity compared to myopia, and anisometropia negatively affects stereopsis when compared to isometropia. These findings highlight the importance of early detection and appropriate correction of refractive errors in children to prevent impairment of binocular vision. Routine screening of stereopsis should be incorporated into pediatric ophthalmic evaluation to ensure optimal visual development.

## Introduction

Stereopsis is defined as the perception of depth and is the highest level of binocular function, often present with good visual acuity. Stereoscopic vision is one of the most important properties of the visual system, which can have an impact on learning capabilities, academic performance, and quality of life, and poor stereopsis can be associated with refractive error, strabismus, amblyopia, anisometropia, etc. Stereopsis, an indicator of binocular vision, can be adversely affected by anisometropia, amblyopia, vergence dysfunction, convergence insufficiency, and uncorrected refractive errors, which disrupt fusion, accommodation, and ocular alignment, thereby reducing depth perception and stereoacuity. Therefore, appropriate refractive correction, detailed clinical assessment, and evaluation of binocular vision parameters, such as convergence, phorias, and accommodative functions, are essential for accurate assessment and preservation of stereopsis [1,2]. Any deficit in stereopsis can affect precision movements, grasping, and sense of distance, which may have a negative impact on their learning capabilities, academic performance, job opportunities, and upcoming quality of life. Stereopsis is important in children for performing fine motor tasks, in sports, and in certain occupations [3,4]. This study assessed the level of stereopsis among children with refractive errors to determine the prevalence of subnormal stereoacuity in the pediatric population. It also aimed to evaluate the association of stereopsis with different types of refractive errors in Central India and to identify factors influencing stereopsis in children.

## Materials and methods

Study design and setting

This was a hospital-based cross-sectional study conducted at the All India Institute of Medical Sciences (AIIMS), Nagpur, Central India, from October 2023 to September 2024. Approval was obtained from the Institutional Ethics Committee, and the study adhered to the principles of the Declaration of Helsinki. A cross-sectional design was chosen as it allows simultaneous assessment of stereopsis and refractive status in a defined population at a single point in time. This design is suitable for estimating the prevalence and pattern of reduced stereoacuity and for evaluating its association with different types of refractive errors in a clinical setting. It is also efficient, less time-consuming, and practical for pediatric populations.

Study population

Children aged 5-17 years with previously diagnosed refractive errors and a history of spectacle use for at least one month were included in the study. Participants were recruited using consecutive sampling from patients attending the ophthalmology outpatient department.

Inclusion Criteria

The following are the inclusion criteria: age between 5 and 17 years, previously diagnosed refractive error, and spectacle use for at least one month.

Exclusion Criteria

The following are the exclusion criteria: latent or manifest strabismus, amblyopia, ocular pathologies affecting vision, and poor compliance with examination procedures.

Sample size determination

The sample size was determined based on previous cross-sectional studies assessing stereopsis in children with refractive errors. Using the standard formula for prevalence studies (n = Z²p(1−p)/d²), and assuming an expected prevalence of abnormal stereopsis of 70%, 95% confidence level, and 10% absolute precision, the minimum sample size was calculated to be approximately 81. To enhance statistical reliability and allow subgroup analysis across different refractive error types, a total of 120 participants were included in the study.

Data collection procedure

All participants underwent a comprehensive ophthalmic evaluation, including visual acuity assessment using Snellen's chart for distance (6 m) and Jaeger chart for near vision (30 cm), slit-lamp biomicroscopy for anterior segment examination, posterior segment evaluation, and ocular motility assessment. Refraction and categorization of refractive errors were done. Objective refraction was performed using both dry and cycloplegic retinoscopy, followed by subjective refraction to determine best-corrected visual acuity. Retinoscopy was performed by a single trained optometrist under standardized conditions to minimize inter-observer variability. Dry retinoscopy was conducted in a semi-dark room at a working distance of 66 cm using a streak retinoscope. Cycloplegic retinoscopy was performed after instillation of 2% homatropine hydrobromide eye drops, with assessment carried out 45 minutes later to ensure adequate cycloplegia. The reflex was evaluated in both principal meridians to achieve neutrality, and the final refractive assessment was based on cycloplegic findings. Refractive errors were categorized based on spherical equivalent (SE) and cylindrical power as follows: myopia: SE ≤ -0.50 diopters (D), hypermetropia: SE ≥ +0.50 D, and astigmatism: cylindrical error ≥ 0.75 D. Spherical equivalent was calculated as sphere + (cylinder/2). In cases with mixed refractive errors, classification was based on the predominant component. Assessment of stereopsis was carried out. Stereoacuity was assessed using the Randot stereotest with participants wearing polarizing glasses over their spectacles. Stereopsis was categorized as follows: normal: 10-60 seconds of arc, subnormal: 61-120 seconds of arc, moderate: 121-240 seconds of arc, and poor: ≥ 241 seconds of arc. Anisometropia was defined as a significant interocular difference in refractive error. Spherical anisometropia was considered as a difference of ≥ 1.00 diopter (D) in spherical power, while cylindrical anisometropia was defined as a difference of ≥ 1.50 D in cylindrical power between the two eyes. These thresholds were based on previously published studies done by Elamurugan et al. [5]. All examinations were conducted by trained optometrists and ophthalmologists using standardized protocols. Retinoscopy was performed by a single examiner to reduce inter-observer variability. However, masking of examiners was not feasible.

Statistical analysis

Data were entered and analyzed using Statistical Package for the Social Sciences (SPSS, version 17.0; SPSS Inc., Chicago, IL). Continuous variables were expressed as mean ± standard deviation (SD), while categorical variables were presented as frequencies and percentages. The association between stereopsis categories and categorical variables (such as type of refractive error and anisometropia) was assessed using the chi-square test. For comparison of continuous variables between groups, appropriate parametric or non-parametric tests (independent t-test or ANOVA) were applied based on data distribution. A p-value of <0.05 was considered statistically significant.

Ethical considerations 

The study was approved by the institutional ethics committee under the IEC number Pharmac/2023/679, after which the study was started. The study was conducted in accordance with the principles outlined in the Declaration of Helsinki. Informed consent in English or the local language, wherever applicable, was obtained, explaining the study, its aim, the option to withdraw from the study, and the guarantee of anonymity.

## Results

The study included a total of 120 patients with refractive errors, with a mean age of 11 ± 2.64 years. Among them, 63 were myopic, 11 were hypermetropic, and 46 had astigmatism. Of the total participants, 65 (54.17%) were males, and 55 (45.83%) were females (Table 1). The mean stereoacuity among all participants was 54.01 ± 32.19 seconds of arc. Anisometropia was present in 19 patients (15.83%).

**Table 1:** Gender distribution

Gender	Frequency	Percentage
Male	65	54.17%
Female	55	45.83%
Total	120	100%

On stereoacuity assessment, normal stereopsis was observed in 61 (96.82%) myopic patients, 43 (93.48%) patients with astigmatism, and 8 (72.73%) hyperopic patients. Moderate-to-poor stereopsis was noted in three (27.27%) hypermetropic patients, whereas only two (3.18%) myopic patients demonstrated subnormal stereopsis. None of the myopic patients exhibited a moderate-to-severe reduction in stereopsis. Although hypermetropic patients showed relatively poorer stereopsis compared to myopic and astigmatic patients, the association between refractive error type and stereopsis was not statistically significant (Table 2) (chi-square = 8.21, df = 6, p = 0.22) (Table 2).

**Table 2:** Stereopsis status in comparison with refractive error

Degree of stereopsis	Myopia (n= 63)	Hypermetropia (n=11)	Astigmatism (n=46)
Normal: 10-60 sec of arc	61 (96.82%)	8 (72.73%)	43 (93.48%)
Subnormal: 61-120 sec of arc	2 (3.18%)	0 (0%)	2 (4.36%)
Moderate: 121-240 sec of arc	0 (0%)	2 (18.18%)	1 (2.17%)
Poor: > 241 sec of arc	0 (0%)	1 (9.10%)	0 (0%)

Gender-wise analysis revealed that normal stereopsis was present in 61 (93.86%) males and 51 (92.72%) females. Subnormal-to-moderate stereopsis was observed in four (6.14%) males and three (5.47%) females, while poor stereopsis was noted in one (1.81%) female patient. There was no statistically significant association between gender and stereopsis (chi-square = 1.22, df = 3, p = 0.75), indicating comparable stereopsis distribution between males and females (Table 3).

**Table 3:** Fusion status in between gender groups

Stereoacuity	Male	Female
Normal	61 (93.86%)	51 (92.72%)
Subnormal	2 (3.07%)	2 (3.66%)
Moderate	2 (3.07%)	1 (1.81%)
Poor	0 (0%)	1 (1.81%)

Furthermore, individuals with isometropia demonstrated better stereopsis; the mean stereoacuity was significantly better in isometropic individuals (48 ± 28.18 arc seconds) compared to anisometropic individuals (60 ± 36.89 arc seconds), and this difference was statistically significant (Table 4) (independent t-test t = 2.31, p = 0.02).

**Table 4:** Comparison of mean stereopsis between isometropia and anisometropic patients

Group	No of patients (n)	Mean Stereopsis (arc sec)	P value
Isometropia	101	48 ± 28.18	
Anisometropia	19	60 ± 36.89	0.02 Significant

## Discussion

Stereopsis represents the highest level of binocular visual function and is responsible for three-dimensional depth perception in humans. Impairment of stereoscopic vision has been associated with reduced performance in activities requiring precise hand-eye coordination and visuomotor integration [3,6]. The present study evaluated the relationship between stereoacuity and various refractive errors. Our findings demonstrated that hyperopic individuals exhibited poorer stereoacuity compared with myopic individuals. Several studies have reported that school-aged children with hyperopia are more likely to exhibit reduced stereoacuity [3,7,8]. Higher magnitudes of hyperopia have also been associated with poorer stereoacuity even among non-strabismic and non-amblyopic preschool children. This reduction in stereoscopic performance is primarily attributed to monocular or binocular retinal blur [9]. Mild degrees of uncorrected hyperopia may be compensated through accommodative effort; however, children with hyperopia greater than 4 diopters often demonstrate increased variability and lag in accommodation, resulting in inaccurate focusing and persistent hyperopic blur. Additionally, hyperopia is associated with a higher risk of amblyopia and strabismus, both of which can further compromise binocular vision and stereopsis [7,10]. Our study findings are consistent with the observations of Eramurugan et al., who reported that myopic individuals demonstrated better stereopsis compared with those with myopic astigmatism and anisometropia [5]. However, Ashtari et al. observed no significant difference in stereoacuity between myopes and hyperopes after providing full refractive correction, suggesting that uncorrected refractive error and associated blur may play a significant role in reducing stereoscopic performance [11].

In the present study, individuals with isometropia demonstrated better stereoacuity compared with those with anisometropia. This finding can be explained by the presence of unequal refractive errors between the two eyes in anisometropia, which may lead to differences in retinal image size, a phenomenon known as aniseikonia. Such disparity in retinal image size can disrupt binocular fusion and impair stereopsis [5]. Importantly, anisometropia has been shown to affect binocular visual development even in the absence of amblyopia. Similar findings were reported by Sethi et al., who observed that subjects with simple refractive errors without amblyopia or strabismus maintained better stereoacuity in cases of isometropia compared with anisometropia [12]. Chanchal et al. further demonstrated that spherical anisometropia greater than 1 diopter was associated with reduced stereoacuity. Experimental studies have also shown that 1 diopter of induced spherical anisometropia can reduce stereoacuity to approximately 57-59 arc seconds, while anisometropia greater than 2 diopters can significantly impair binocular visual function [13]. Campos et al. reported that aniseikonia exceeding 5% may result in loss of stereopsis. Furthermore, aniseikonia may be more pronounced in cylindrical refractive errors compared with spherical errors [14]. In a study evaluating myopic ametropia associated with astigmatism, Yang et al. reported reduced stereoacuity in cases of spherical anisometropia [15]. However, in another similar study, the same authors reported reduced stereoacuity predominantly in hyperopic individuals, while anisometropia associated with spherical or astigmatic refractive errors did not demonstrate a statistically significant reduction in stereoacuity [1].

With respect to gender distribution, the present study demonstrated normal stereopsis in 61 (93.86%) male patients and 51 (92.72%) female patients, with an overall mean stereoacuity of 54.01 ± 32.19 arc seconds. These findings are comparable to those reported by Guo et al., who observed a mean stereoacuity of 50.2 ± 50.6 arc seconds among children [3]. In contrast, Sethi et al. reported a relatively higher mean stereoacuity of 120.60 ± 76.36 arc seconds [12]. Although male participants in our study demonstrated marginally better stereoacuity than female participants, the difference was not statistically significant, suggesting that gender may not have a meaningful influence on stereoscopic vision in individuals with refractive errors. The findings of the present study highlight the importance of evaluating binocular visual function, particularly stereopsis, in addition to assessing visual acuity and ocular alignment. Restoration of optimal binocular vision should be considered an important goal in the management of refractive errors and other visual disorders. Early detection and timely correction of refractive errors in children are therefore essential, as delayed intervention may predispose to the development of amblyopia and compromised binocular visual development [13,16]. Hence, prompt visual rehabilitation during the critical period of visual maturation may help preserve or improve stereoscopic vision and contribute to better overall visual function in children.

This study has certain limitations. Being a hospital-based cross-sectional study in central India, the findings may not be generalizable to the broader population. As it was not a longitudinal study, we were unable to track development and changes in the same individuals for a longer duration. The lack of detailed binocular vision and accommodative assessments, such as fusional vergence ranges, vergence facility, and amplitude of accommodation, which may influence stereopsis outcomes, can also be considered one of the limitations of the study. It is important to note that our study had a relatively smaller sample size, and masking of the examiner was not done, which could have introduced the possibility of observer bias. Considering the limitations of the present study, larger-scale studies are warranted to better determine the extent of reduced stereopsis across different refractive groups.

## Conclusions

The present study demonstrates that hypermetropia and anisometropia are significantly associated with reduced stereoacuity in children, even in the absence of overt strabismus or amblyopia. In contrast, children with isometropia and myopia showed relatively better stereoscopic outcomes. These findings underscore the importance of a comprehensive visual assessment that includes evaluation of stereopsis in addition to visual acuity and refractive status. Early detection and timely optical correction of refractive errors during the critical period of visual development are essential to preserve binocular function and optimize visual outcomes. Nonetheless, routine screening of stereopsis in children with refractive errors is recommended, as early detection and management may help improve stereoscopic function and prevent compromise of binocular vision.
